# When Bowel Motility Goes Awry: Ogilvie’s Syndrome Following Relaparotomy for Intra-abdominal Bleeding After Caesarean Section

**DOI:** 10.7759/cureus.95009

**Published:** 2025-10-20

**Authors:** Leh Ping Sii, Ing Hie Wong, Marcus Kang

**Affiliations:** 1 Obstetrics and Gynaecology, Hospital Sibu, Sibu, MYS

**Keywords:** acute colonic pseudo-obstruction, caesarean section, colonic dilatation, mechanical intestinal obstruction, ogilvie’s syndrome, paralytic ileum, postoperative complication, relaparotomy

## Abstract

Acute colonic pseudo-obstruction (Ogilvie’s syndrome) is a rare but potentially life-threatening postoperative complication characterised by massive colonic dilatation without mechanical obstruction. We report the case of a 40-year-old woman who developed Ogilvie’s syndrome following relaparotomy for intra-abdominal bleeding after an elective caesarean section. This case highlights the diagnostic challenge of differentiating Ogilvie’s syndrome from postoperative ileus and underscores the importance of early recognition to prevent serious complications such as caecal perforation.

## Introduction

Ogilvie’s syndrome represents a rare postoperative disorder that can be life-threatening, presenting with extensive colonic dilatation in the absence of a true mechanical obstruction [1]. Sir William Ogilvie first documented the condition in 1948, when he observed it in two patients with malignancy involving the prevertebral ganglia [2]. The true incidence of Ogilvie’s syndrome is probably underreported, as it is often missed until there is significant abdominal distension, and many subclinical cases may go unrecognised, with patients recovering spontaneously [3]. If the condition is not promptly recognised and managed, it can progress to severe colonic distension with risks of bowel ischaemia and eventual perforation [3, 4]. Furthermore, it is often misdiagnosed as paralytic ileus because of overlapping clinical features, particularly in postoperative patients. Paralytic ileus is a neurogenic condition that predominantly affects the small intestine [5]. In most cases of Ogilvie’s syndrome, dilatation is usually confined to the proximal large bowel, especially the caecum, ascending colon, and sometimes up to the splenic flexure [6]. Pelvic procedures, particularly caesarean deliveries, are the surgeries most frequently associated with the onset of acute colonic pseudo-obstruction, and such cases are estimated to contribute to about one-tenth of the overall reported incidence [7]. Mortality is influenced by several factors, including patient age, caecal diameter, timeliness of intervention, and the condition of the affected bowel. With prompt treatment, the fatality rate is about 15%, whereas it rises to 36-44% once perforation or ischaemia occurs [8]. We report the case of a 40-year-old woman who developed Ogilvie’s syndrome following relaparotomy for intra-abdominal bleeding after an elective caesarean section.

## Case presentation

A 40-year-old woman with epilepsy, well-controlled type II diabetes mellitus, chronic hypertension, and fatty liver underwent elective repeat caesarean section with bilateral tubal ligation. The surgery was uncomplicated, but she developed postpartum haemorrhage requiring relaparotomy, during which 2.4 L of haemoperitoneum was evacuated, with no obvious bleeders identified despite thorough exploration, and Hayman sutures were applied as uterine atony was also noted intraoperatively.

Twenty-four hours after operation, she developed progressive abdominal distension and electrolyte imbalance (hyponatraemia, hypokalaemia, hypomagnesaemia), despite still being able to pass flatus and have bowel opening. Imaging revealed dilated small and large bowel loops without a transitional point. A diagnosis of Ogilvie’s syndrome was made after excluding peritonitis and mechanical obstruction.

She was managed conservatively with nil by mouth, nasogastric decompression, intravenous fluids, electrolyte correction, broad-spectrum antibiotics, and erythromycin as a prokinetic. Her condition gradually improved, and she was discharged well on day 14 post-caesarean section.

Blood investigations revealed deranged electrolytes with hyponatraemia, hypokalaemia, and hypomagnesaemia, while calcium levels remained normal. White cell count was elevated. Blood gas analysis showed no acidosis. Lactate levels were mildly elevated. Renal profile was within normal limits (Table 1). Haemoglobin dropped from 110 g/L preoperatively to 58 g/L at 24 hours postoperatively, then rose to 102 g/L at 37 hours post transfusion, indicating significant blood loss followed by partial recovery after transfusion. Urine creatinine was compared with peritoneal fluid, which was not suggestive of uroperitoneum. 

**Table 1:** Laboratory investigations Laboratory values are presented with corresponding reference ranges. Abnormal values are highlighted as high or low. WCC: white cell count.

Parameter	Patient Value	Reference Range	Interpretation
Sodium	132 mmol/L	135-145 mmol/L	Low
Potassium	2.8 mmol/L	3.5-5.0 mmol/L	Low
Magnesium	0.66 mmol/L	0.7-1.0 mmol/L	Low
Calcium	2.4 mmol/L	2.1-2.6 mmol/L	Normal
WCC	17.7 x 10^3^	4-11 x 10^3^ /mL	High
pH	7.38	7.35-7.45	Normal
HCO_3_	23.7 mmol/L	22.0-26.0 mmol/L	Normal
Base excess (BE)	-1.3	-2 to +2	Normal
Lactate	1.8-2.2 mmol/L	<2.0 mmol/L	Mildly high
Urea	5.0 mmol/L	2.5-7.0 mmol/L	Normal
Creatinine	44 mmol/L	44-106 mmol/L	Normal

**Figure 1:**
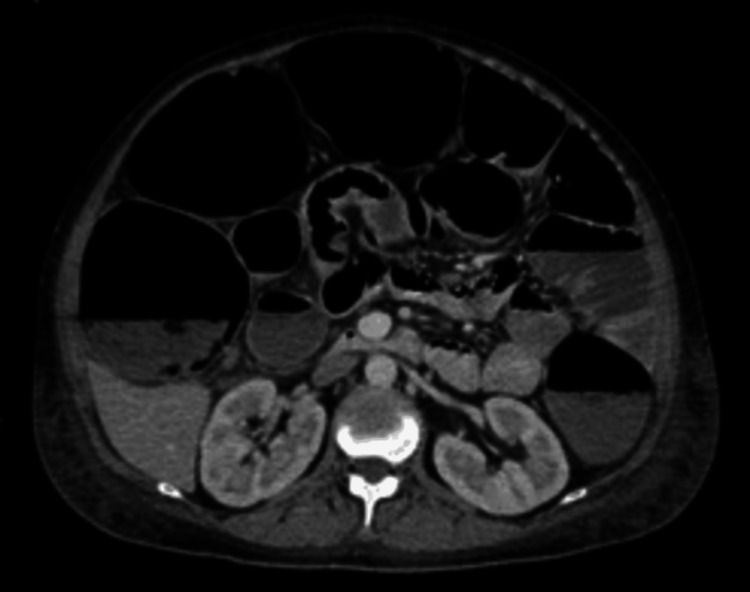
Contrast-enhanced computed tomography (CECT) of the abdomen showed diffuse small bowel dilatation measuring up to 4.3 cm and large bowel dilatation measuring up to 7.5 cm, with no identifiable transitional point.

**Figure 2:**
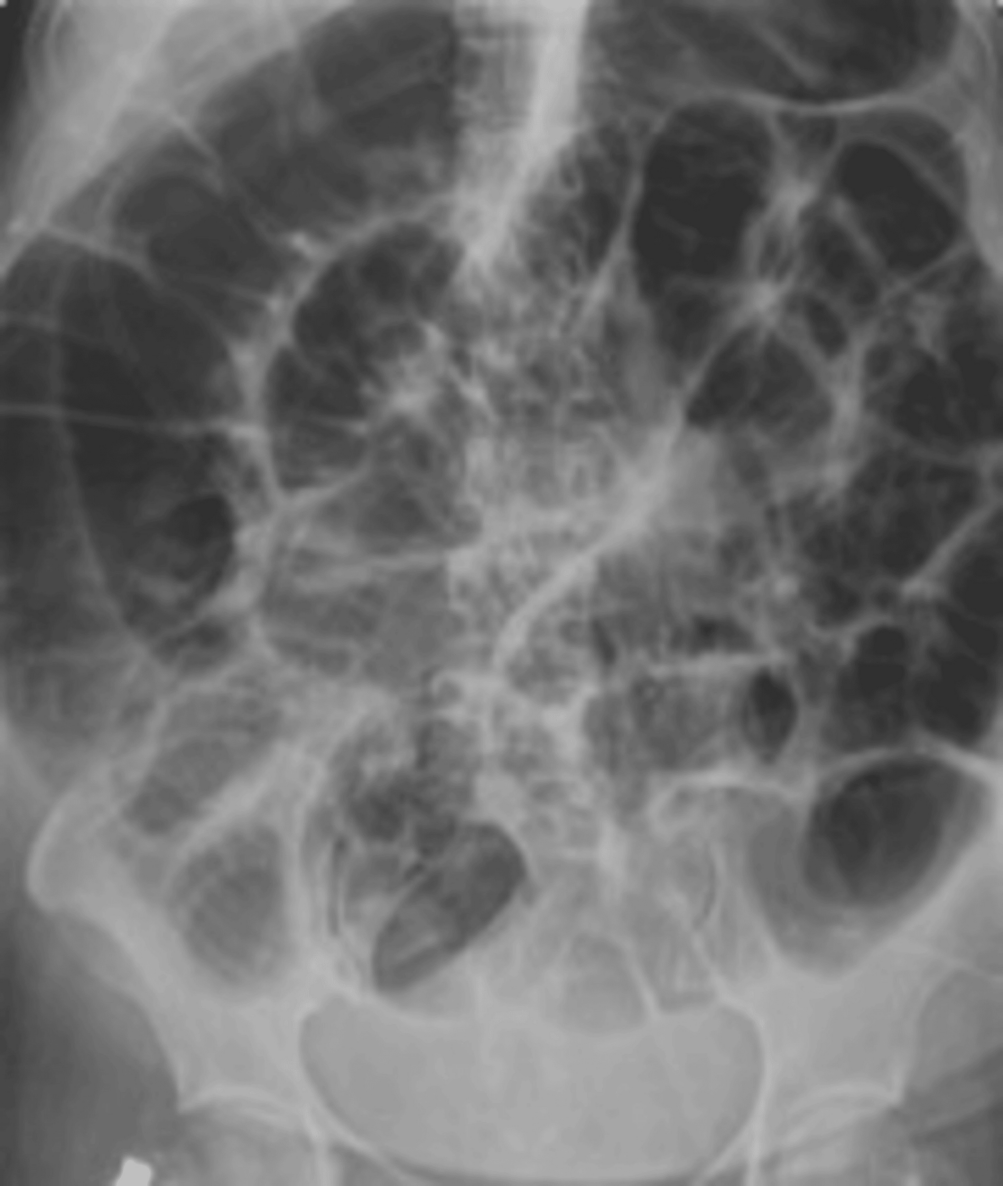
Abdominal radiograph showing diffuse colonic dilatation, most prominent in the cecum, consistent with acute colonic pseudo-obstruction (Ogilvie's syndrome).

Abdominal X-ray showed dilated colons (see Figure 1). CECT abdomen noted diffuse small bowel dilatation measuring up to 4.3 cm and large bowel dilatation measuring up to 7.5 cm, with no identifiable transitional point. Also noted were focal segmental distal and terminal ileal wall thickening, a dilated appendix, and lymph nodes, which may represent an infective or inflammatory process. There was no pneumoperitoneum or fluid collection (see Figure 2). 

## Discussion

Ogilvie’s syndrome is an uncommon but potentially serious condition that may follow any pelvic operation. The underlying mechanism is believed to involve disruption of autonomic regulation, with sympathetic overactivity and diminished parasympathetic activity [7]. Several risk factors have been identified, including recent pelvic surgery, metabolic or electrolyte disturbance, systemic infection, and the use of certain medications such as opioids, anticholinergics, phenothiazines, corticosteroids, and calcium channel blockers [9].

In its early stages, the condition can be difficult to distinguish from paralytic ileus, as both present with abdominal distension and impaired bowel function. However, preservation of small bowel activity with dilatation confined to the colon, along with the potential for rapid caecal enlargement, should raise suspicion of Ogilvie’s syndrome [6]. The onset of symptoms usually occurs within the first two days, with a median of 48 hours, although cases have been reported as early as six hours postpartum [7]. It is mainly manifested as marked abdominal distension [10], with abdominal pain described as a dull, crampy sensation with no specific localisation, typical of hollow viscus distension, which may then progress to localise over the right iliac fossa, indicating impending rupture of the caecum [11].

Although caecal perforation is relatively uncommon, occurring in only 1% to 3% of patients, it carries a high risk of death, with a mortality rate between 50% and 71%, compared with around 8% in those without perforation [4]. Low-grade pyrexia, leucocytosis, and rising CRP levels are likely to represent worsening acute colonic pseudo-obstruction [7]. Diagnosis requires exclusion of mechanical obstruction, usually with contrast-enhanced CT, which shows proximal colonic dilatation with a transition point at the level of the splenic flexure [12].

Management is guided by the degree of colonic dilatation and the patient’s overall condition. In the absence of perforation, conservative treatment is often effective, especially when caecal dilatation is less than 10 cm [11]. This typically includes supportive therapy, correction of metabolic disturbances, and withdrawal of offending drugs [13].

When conservative measures fail, neostigmine can be considered, provided contraindications such as mechanical obstruction, bronchial asthma, or suspected perforation are excluded. Administered intravenously, neostigmine enhances colonic motility through acetylcholinesterase inhibition. A prompt response, often passage of flatus and relief of abdominal distension, is observed in most cases, with a reported success rate of up to 85% [14]. Colonoscopic decompression remains a further option if drug therapy is unsuccessful, although recurrence may occur [11].

## Conclusions

The most widely accepted explanation for Ogilvie’s syndrome is an autonomic imbalance, with excessive sympathetic drive and reduced parasympathetic activity, most often seen in critically ill or postoperative patients. Without prompt recognition and treatment, the condition is associated with considerable morbidity, and mortality remains substantial. A pragmatic and structured management approach is therefore essential to achieve a favourable outcome. Early recognition and conservative management may facilitate full recovery and prevent the need for invasive intervention. If conservative therapy fails, medical treatment such as intravenous neostigmine, followed by colonoscopic decompression when indicated, can be considered as the next steps. Definitive surgical intervention is generally reserved for complicated cases, particularly those with bowel perforation or evidence of ischaemia.
